# Trajectories of Episodic Disability in People Aging with HIV: A Longitudinal Qualitative Study

**DOI:** 10.1177/2325958218759210

**Published:** 2018-02-21

**Authors:** Patricia Solomon, Kelly Kathleen O’Brien, Stephanie Nixon, Lori Letts, Larry Baxter, Nicole Gervais

**Affiliations:** 1 School of Rehabilitation Science, McMaster University, Hamilton, Ontario, Canada; 2 Department of Physical Therapy, University of Toronto, Toronto, Ontario, Canada; 3 Community HIV Volunteer, Halifax, Nova Scotia, Canada

**Keywords:** HIV, episodic disability, longitudinal analysis

## Abstract

People living with HIV may experience disability which is episodic in nature, characterized by periods of wellness and illness. The purpose of this longitudinal qualitative study was to understand how the episodic nature of HIV and the associated uncertainty shape the disability experience of older adults living with HIV over time. Fourteen men and 10 women who were HIV positive and over 50 years (mean age: 57 years; range: 50-73) participated in 4 interviews over 20 months. Longitudinal analyses of the transcribed interviews identified 4 phenotypes of episodic disability over time: decreasing, increasing, stable, or significant fluctuations. Although all participants experienced uncertainty, acceptance and optimism were hallmarks of those whose phenotypes were stable or improved over time. Understanding a person’s episodic trajectory may help to tailor interventions to promote stability, mitigate an upward trajectory of increasing disability, and increase the time between episodes of illness.

## Introduction

Over the past decade, there has been increased attention to aging as an important characteristic of the HIV epidemic. With advances in combination antiretroviral therapy (cART), people living with HIV (PLWH) may survive for 30 to 50 years after infection.^1,2^ The consequences for those aging with HIV include the long-term impact of living with the virus and the adverse effects of cART, coupled with the natural processes of decline and deterioration associated with aging. This is further complicated by the decrease in the ability of the immune system to respond to pathogens or immunosenescence as people age.^3^ There is an increased incidence of bone and joint disorders, cardiovascular disease, metabolic syndrome and cancer, earlier onset of frailty, and an increased risk of neurocognitive decline among those aging with HIV.^4^ Health consequences associated with these comorbidities can be confounded by the aging process.^5^ Furthermore, adults aging with HIV can experience the added complexity related to the social determinants of health and the double stigma of ageism and living with HIV. These factors have an additive and synergistic effect on increasing the risk of numerous common comorbidities.^6^

The combination of physical, mental, and social health-related consequences associated with HIV, aging, and comorbidities may result in disability.^7^ The disability experienced by adults with HIV is recognized as episodic in nature, often related to fluctuating periods of wellness and illness.^7,8^ The Episodic Disability Framework was derived from the perspectives of 38 PLWH. It spans physical, mental, emotional, and social life domains including challenges of uncertainty or worrying about future health and includes 4 dimensions of disability: symptoms and impairments, difficulties with day-to-day activities, challenges to social inclusion, and uncertainty. The framework also highlights the importance of understanding how extrinsic contextual factors (eg, social support, stigma) and intrinsic contextual factors (eg, living strategies, personal attributes such as age and comorbidity) exacerbate or alleviate disability. This framework identifies uncertainty as a key dimension of disability and incorporates the episodic nature of disability over time. It also describes in detail the living strategies that individuals use to alleviate disability.^8^

The Episodic Disability Framework was seminal in increasing our understanding of the consequences of HIV and its associated treatments. However, the episodic nature of the disability experienced by those aging with HIV is not well understood. Studies have been cross-sectional in design, which limit the ability to understand the profile and consequences of disability over time. Living with an episodic illness brings unique challenges associated with the unpredictable nature of HIV. As with aging, episodic disability is a temporal process and the consequences and contributions to disablement are best illuminated through longitudinal study.

Qualitative longitudinal study design allows for an increased understanding of health-related challenges experienced over time and of the strategies individuals use to make sense of the past and navigate their future.^9^ This approach can provide insights into the contextual factors influencing disability, an important consideration for identifying treatment strategies aimed to reduce disability and improve overall health. The methodology can complement quantitative studies examining change in health status over time and provide more nuanced insights. The purpose of this study was to understand how the episodic nature of HIV and the associated uncertainty shape the disability experience of adults aging with HIV over time.

## Methods

We conducted a longitudinal qualitative study interviewing older adults living with HIV on repeated occasions to explore changes in disability over time. Through this method, we sought a deeper understanding of *how and why* change occurs and the factors accounting for the changes over time.^9^ This research was approved by research ethics boards of McMaster University and University of Toronto.

### Study Procedures

We conducted a series of 4 semi-structured interviews with older adults living with HIV, at 5-month intervals. Participants were recruited through HIV community service organizations in Southern Ontario, Canada, by placing pamphlets on-site, on websites, and distributing via e-blasts. In addition, we recruited through the website of *Realize* (formerly the Canadian Working Group on HIV and Rehabilitation).

Individuals were eligible to participate if they were 50 years of age or older and able to read, speak, and understand English. Because evidence suggests that the presentation of illness in PLWH varies between individuals in their fifth decade and those in their sixth decade,^10^ we sought to recruit across a range of age groups. We also sought balanced representation of men and women. Interviews took place in accessible locations, typically HIV community service organizations that were mutually agreed upon by participants and investigators.

We used the Episodic Disability Framework to guide the semi-structured interviews.^8^ During the first interview (time 1), we asked participants to describe their health challenges including (1) physical, cognitive, mental, and emotional symptoms and impairments; (2) difficulties carrying out day-to-day activities; and (3) challenges to social inclusion. These challenges were then explored in detail, probing the episodic nature of the challenges, the uncertainty or worrying about future health, and the intrinsic and extrinsic contextual factors affecting the challenges. We also explored the living strategies and social supports that participants used to address the health challenges (and uncertainty) related to aging with HIV. To understand the episodic nature of HIV, in the subsequent interviews, we explored the specific health challenges identified in time 1 and asked the participant to consider what changes occurred (if any), how these occurred, and how these changes affected their functioning, disability, and health. Our longitudinal study design allowed for emergent themes to be discussed with participants over time. We explored specific challenges identified in previous interviews in subsequent interviews, and participants also were able to identify new challenges that arose.

At time 1, participants completed a demographic questionnaire to describe their personal characteristics and the HIV symptom severity index.^11^ Participants received an honorarium at the completion of each interview.

### Analysis

Interviews were audio recorded and transcribed verbatim. Transcripts were quality checked by the interviewer. The transcripts were entered into NVIVO 9^12^ to manage and organize the data.

Longitudinal analyses included summary and comparison of data both cross-sectional and longitudinal.^13^ After the completion of the time 1 interviews, we developed a codebook to guide our analysis using an open coding procedure^14^ of 3 transcripts by 2 investigators. We derived the final codebook by reconciling discrepancies and combining codes. The remaining time 1 transcripts were then allocated to 1 of 3 pairs of investigators to code. Using the coded transcripts, each pair developed an in-depth cross-sectional summary profile for each participant.

In order to examine the episodic nature of each participant’s disability over time, we coded the data using a structured approach guided by the Episodic Disability Framework. Each investigator reviewed the time 2 transcript of a participant and documented any changes that had occurred since time 1 using the categories in the Episodic Disability Framework. New challenges or symptoms that emerged were noted. We repeated this same process with the time 3 and time 4 transcripts. Next, we developed an in-depth longitudinal summary profile that described the episodic nature of disability experienced by each participant over time. Summary profiles for each participant were completed independently by 2 investigators and amalgamated into 1 overall profile. In the final step, we compared the longitudinal summary profiles across participants to document similarities and differences in the episodic nature of disability experienced over time. This comparison resulted in the identification of common trajectories of episodic disability.

We then developed graphic representations of each phenotype based on an individual participant, with the *y*-axis representing the level of disability experienced and the *x*-axis representing the health-related challenges. We plotted sources of disability that related to the most significant challenges experienced by each of the 4 participants over the course of the interview. These were qualitatively derived based on the level of disability associated with each condition or symptom described at the initial interview. For subsequent interviews, we used time 1 as a baseline to reflect the participant’s views on whether the condition or symptom had improved, worsened, or remained the same. New health challenges were graphed as these arose. We also depicted any fluctuations between time points that the participant described.

## Results

Twenty-four participants (14 men and 10 women) participated in the study, each of whom completed 4 interviews over 20 months for a 100% retention rate. See Table 1 for disease and demographic characteristics of the participants.

**Table 1.** Demographic Characteristics of Study Participants.^a^

Characteristic	Mean (min-max) or Number (%)
Gender	
Male	14 (58%)
Female	10 (42%)
Mean age	57 years (50-73)
Mean time since diagnosis	18 years (6-30)
CD4 count >400	19 (79%)
Undetectable viral load	18 (75%)
HIV symptom index	
Median number of symptoms present	12.5/20 (IQR^b^: 9-18)
Median number of bothersome symptoms present	10.5/20 (IQR^b^: 6.25-13.5)
Marital status	
Single	12 (50%)
Married or living with partner	6 (25%)
Divorced or widowed	6 (25%)
Financial situation	
Difficulty making ends meet	7 (29.2%)
Just enough to get along	12 (50%)
Comfortable	4 (16.7%)
Estimated gross family annual income^c^	
Less than $10 000	4 (16.7%)
$10 000 to less than $40 000	15 (65.2%)
$40 000 to less than $70 000	2 (8.7%)
$70 000 or more	2 (8.7%)
Current employment status	
Work full- or part-time	5 (20.8%)
On disability	13 (54.2%)
Retired	4 (16.7%)
Unemployed, seeking work	2 (8.3%)

^a^N = 24.

^b^Inter Quartile Range.

^c^CAD.

### Trajectories of Episodic Disability

**Figure 1.**
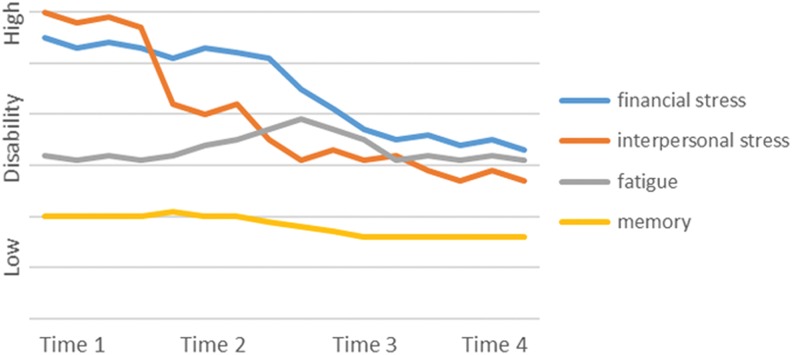
Participant A. Example of decreasing disability over time.

**Figure 2. fig2-2325958218759210:**
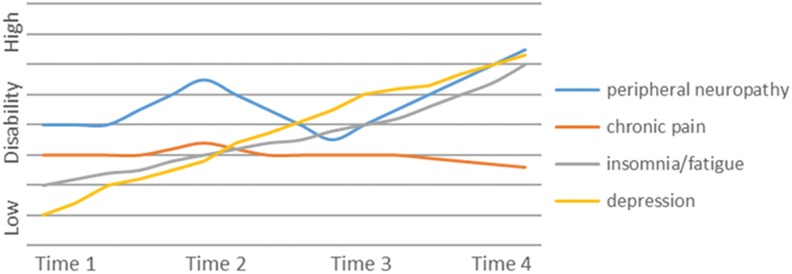
Participant B. Example of increasing disability over time.

**Figure 3. fig3-2325958218759210:**
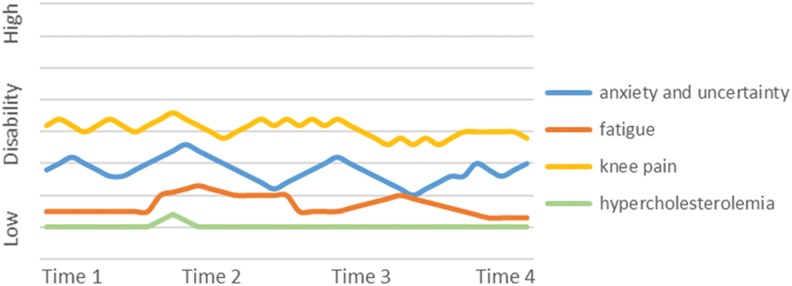
Participant C. Example of stable over time.

The episodic disability experience varied among participants. We identified 4 main “phenotypes” that characterized the trajectories of living with episodic disability that included decreasing disability over time, increasing disability over time, stable disability over time, and significant fluctuations in disability over time. We provide an example and graphic representation of the trajectory of 1 participant for each phenotype in Figures 1 [Fig fig2-2325958218759210][Fig fig3-2325958218759210]to 4.

**Figure 4.**
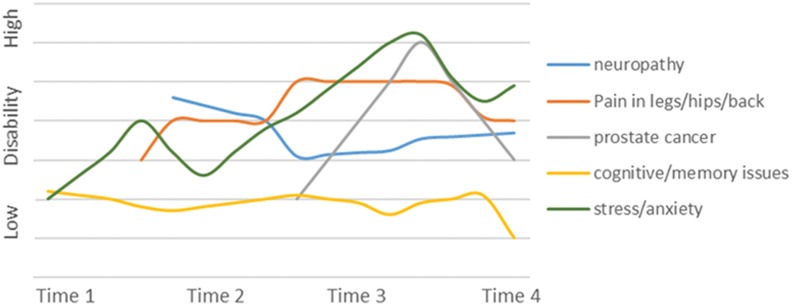
Participant D. Example of significant fluctuations in disability over time.

### Decreasing Disability over Time

Four participants experienced improvements in their disability over time. Although they had many health challenges (or disability), they did not experience any major new symptoms and were engaged in ongoing strategies that improved their health by increasing activity levels, engaging in new pursuits, and keeping a positive frame of mind. These participants explained that they accepted that aging with HIV came with limitations but they had “learned to work around it.” One man acknowledged that there would be fluctuations in his illness and disability but attributed these primarily to changes in the weather as he could not be physically active in the winter and the cold and darkness affected his mood. One woman was not optimistic about her health during the first 2 interviews and described how the challenging family dynamics affected her health. She developed strategies that resulted in improvements over time, “I’ve learned that I have resilience…I think I’m going to get a new mindset and a new direction.” Another man described significant fatigue and lack of energy; he recognized that his life was filled with uncertainty but he described having a sense of control over certain issues and planned ahead to have his “house in order” in case his health took a turn for the worse. He described his problem-solving when encountering a new problem in order to reduce disability and improve his overall health:

(there is a) long stretch of time where there’s no episodes, and it could be incredible fatigue, or it could be nausea, for no apparent reason, and sometimes you have to do a problem solving thing where, “is it something that I’m eating? Is it causing the nausea? Or is it the meds?” You got to do like Sherlock Holmes: “am I on a new medication? Could that be the reason?” So, like I say, a lot of times you deal with it yourself, and if you can’t solve it, that’s when you go for professional help. But it’s the nature of the beast.

Trying to remain optimistic and maintaining a healthy lifestyle characterized this group who experienced decreases to disability over time.

#### Example of decreasing disability over time

This participant is a 65-year-old single gay man diagnosed with HIV 20 years ago (Figure 1). He is a retired health professional who worked throughout his life and was able to retire on a full pension. He is active in the community, volunteering for HIV community organizations and his church, though he speaks of drawing back from his time commitments in the future. He maintains a healthy lifestyle through exercise and avoiding smoking and alcohol and has a strong supportive network of family and friends. His primary challenges include fatigue and decreased energy though he developed strategies and “learned to work around it.” He reports comorbid heart and thyroid disease and visual challenges that limit mobility. He has some cognitive issues primarily related to memory. He experiences uncertainty related to his fatigue levels, his ability to manage household tasks, and about the source of his memory challenges. When asked how he manages his episodic challenges, he is optimistic and discusses self-management strategies that successfully decreased the disability he is experiencing over time.

### Increasing Disability over Time

Six participants experienced a continual worsening of their health challenges over time with no or minimal relief or periods of improvement. Although fluctuations in health and disability occurred on a daily basis, the overall trajectory was one of deterioration or an increase in disability over time as expressed by one woman who reported that “it’s worse. It gets worse every day.” For some this was a very slow decline, which they attributed to aging, often describing changes to strength, energy, balance, stiffness, and mobility. Participants described increased disability attributed to changes in their physical, cognitive, and emotional health. They did not experience periods of “good” health as this man noted: “Well it’s been cycling down, it hasn’t cycled up.” Participants in this group described uncertainty about the future related to disclosure; living with pain, fatigue, and memory issues; speaking about depression; and the difficulties in finding joy in life. One man stated, “I don’t think I’m fun to be with anymore, ‘cause I don’t like being me.” The aging process seemed to compound the HIV-related challenges. One woman entered an HIV hospice for 2 months during the study. Some struggled with disclosure and this limited the support they were able to get to deal with new challenges or exacerbations of existing challenges. These participants also commented on the effects of weather on mobility and activity levels, with one woman expressing that she “hates winter with a passion.” This group was engaged in passive coping strategies as expressed by this woman, “When you get up in the morning, you’re hoping and praying that you’re going to have a good day.”

#### Example of increasing disability over time

This 52-year-old woman is a widowed immigrant to Canada from South America who was diagnosed with HIV 25 years ago (Figure 2). She worries about disclosure so minimizes social relationships and as a result finds herself socially isolated. She maintains daily contact with her son who resides in the same city. She describes experiencing peripheral neuropathy and paresthesia, chronic pain, insomnia, arthritis, fatigue, and lipodystrophy. She also has depression and difficulty with memory and focusing attention. Although at the initial interview she describes herself as “healthy,” over time focusing on her comorbidities comprise a large portion of her daily experiences and are sources contributing to her pain and associated uncertainty. This uncertainty limits her engagement in activities and her social participation. Although her fatigue, pain, depression, and neuropathy fluctuate, the trajectory is one of increasing disability over time.

### Stable over Time

Eight participants showed little variation in their disability over time. Participants in this group had multiple comorbidities and did not describe themselves as having “good health.” However, when participants who were stable experienced new challenges, they were able to engage in strategies that helped to mitigate further disability. One 75-year-old man with limited mobility used a wheelchair and a walker; he had diabetes and experienced episodic fatigue, back pain, and many physical, emotional, and cognitive challenges, yet felt he was aging successfully. These participants identified that maintaining their health involved commitment, as stated by this woman, “It’s a lot of work to being on top of things.” Although there were many sources of uncertainty, either these remained stable over time or the participant didn’t dwell on these and were “going with the flow.” Many were actively trying to maintain and improve their quality of life, working at setting realistic expectations and engaging in meaningful activities. Some had spent time trying to understand and adapt to their limitations. There was a sense of acceptance of aging as part of their overall picture of health as this man stated, “you have to adjust to where you are at. I’m 74 now and I intend to be 80 at least, so I adapt to it as I go along.” For some, being stable in health and disability was deemed to be an “improvement” from previous stages of their lives. One woman noted her long struggle with depression and how she “would fight tooth and nail to try to not go back there again.” Although stable participants experienced fluctuations in their health challenges, the overall picture was one of maintaining the level of their health and disability over time. This man stated, “I had my ups and downs, but more ups than downs, so honestly I’m doing really good.” Similar to the other groups, weather affected activities and social engagement through increases in joint pain and fatigue and changes in mood, with this woman noting, “The winter time you just go right down. I do get blue in the winter time. I do.” Acceptance and a positive attitude were prevalent in the stable phenotype. This woman stated:

I keep believing in my health (that) I’m going to get a little bit better. I mean, maybe I won’t get a lot better; I’m never obviously going to get back to what I was 5 years ago.

#### Example of stable over time

This 59-year-old woman lives with her husband and her college-aged children (Figure 3). She has been living with HIV for 6 years. Her health remains relatively stable; when she does face health challenges, she uses self-care techniques and health-care services to return to her baseline level. She is actively engaged in work, volunteering, and social activities in the community. She faces some challenges in her social participation (eg, getting laid off from a job) but uses her skills to compensate (ie, finding a new job). Uncertainty is evident as she worries about comorbidities, disclosure, health provider knowledge, future housing, finances, and employment. She complains of fluctuations in knee pain which she manages through planning her walking routes and exercise. She also has strategies to manage her fatigue level and hypercholesterolemia which remain stable over the duration of the study.

### Significant Fluctuations in Disability over Time

Six participants experienced significant fluctuations in their health challenges and related disability over time. The magnitude of the fluctuations was associated with greater levels of uncertainty. Participants were diagnosed with new illnesses during the course of the study and experienced new symptoms over time including cancer, gastrointestinal issues, insomnia, memory and concentration issues, arthritis, peripheral neuropathy, and depression. The uncertainty of participants’ health status often curtailed volunteer, work, and social activities, with the level of disability (ie, engagement in day-to-day activities and social participation) fluctuating with the severity of the health condition. Occasionally, participants could identify a specific trigger of an increase in disability; for example, one man’s job loss led to financial troubles, loss of his supportive network, and challenges with his partner. Uncertainty about whether the cause of the health challenge was related to HIV or aging was evident in this group, with one man saying, “It’s become more difficult, the more I age, to separate what’s aging and what’s HIV.” Some of the participants learned to live with the unpredictable nature of their illness; while others focused on this to the detriment of their overall health and did not plan for the future. As with the other groups, the influence of weather on the episodic nature of disability was identified with issues related to increased neuropathic pain, exacerbations of arthritis, and decreased activity levels reported in the winter months, with one man stating that the bad weather had him “wanting to stay in bed and pull the covers over his head.”

#### Example of significant fluctuations (improvements and deterioration) in disability over time

This 61-year-old gay man is a long-term survivor diagnosed with HIV 29 years ago (Figure 4). He continued working for many years but eventually left the workforce due to an illness. While he feels he is no longer employable, he is an active volunteer in the HIV community. He has an elderly mother who lives close to him whom he feels responsible for, but otherwise he describes himself as socially isolated. He experienced many new physical and emotional challenges over the course of the study and was diagnosed with prostate cancer. He appeared highly anxious and pessimistic about his future health. There were significant fluctuations in his physical health challenges related to neuropathy, pain, sciatica, emotional distress, and memory over time. His social participation was episodic, varying with his physical and emotional health.

## Discussion

This study provided insight into the episodic nature of disability by following older adults living with HIV over a 20-month time frame. Despite considerable variation among participant experiences, overall we identified 4 phenotypes to conceptualize the episodic nature of disability. Identifying patterns in experiences may assist clinicians in tailoring management approaches by working with adults aging with HIV to help them understand triggers of episodes and recognize successful strategies to mitigate or prevent disability aging with HIV.

Uncertainty about future health may result from dealing with an unpredictable illness^15^ and has been postulated as being at the center of disability experienced by older PLWH.^16^ Although participants across all phenotypes experienced some degree of uncertainty, acceptance and optimism were hallmarks of those whose experiences were stable over time and those who improved over time. These qualities are also related to resilience and hardiness, positive concepts of aging found in the general population^17^ and in those living with HIV.^18^ Positive psychological traits such as optimism have been found to be more predictive of successful aging compared to physical or biological markers of HIV disease.^19^ Promoting problem-solving and positive reframing in long-term HIV survivors have also been identified as important to help adults with HIV cope with their challenges.^20^

Self-management strategies support the development of problem-solving and are recognized as important in dealing with chronic conditions.^21^ Morris et al^22^ noted the challenges for patients in using self-management strategies to deal with multiple comorbidities. Their qualitative study of 30 individuals living with chronic illness revealed that respondents underwent a dynamic process of reprioritizing their conditions and management practices. Respondents were able to prioritize more effectively when they could see how they could use existing self-management practices on new conditions that arise. This suggests an important role for health providers in helping PLWH see the transferability of their existing skills and practices in the management of episodic illness.

Weather was an unexpected trigger for episodes of illness across all trajectories. Participants spoke of decreased activity levels during the winter due to cold and fear of falling, of winter having an effect on their mood, and of becoming more socially isolated during the winter months. The Canadian winter over which the interviews occurred was particularly harsh and this negative influence may not be identified to the same extent in milder years with less snow. Regardless, identifying winter as a trigger of episodes suggests that health providers be proactive and provide strategies to promote mobility and social participation and mitigate depression in older PLWH who may be at risk.

Although our findings describe the experiences of PLWH 50 years of age or older, it is likely that some of the experiences are not unique to older adults. Participants attributed some of their health challenges to aging. As in other research, they also recognized the presence of uncertainty as to whether their health challenges were related to aging or HIV,^15^ or a combination of the two. Positive living strategies such as self-acceptance and self-management can provide PLWH with a sense of control through combating uncertainty.^15^ This reinforces the needs for health professionals to provide support on how to mitigate uncertainty with PLWH. To the extent that there are multiple sources of uncertainty across all ages, assessment of uncertainty should not be limited to those 50 years and older living with HIV.

Graphing their experiences highlights the complexities of the participants’ disability over time and can provide further insights to help PLWH see their own patterns and living strategies. Getting PLWH to graph their symptoms over time may facilitate discussions on successes and challenges and be a useful tool to promote self-management strategies. The goal would be to increase the length of time between episodes, promote downward trajectories (improving health), and minimize disability.

The methodology can complement quantitative studies examining change in health status over time and provide more nuanced insights. An advantage of our qualitative approach was the in-depth discussion of sensitive issues that was enabled by the development of an ongoing relationship between the participants and the interviewer.^23^ Another strength of our study was the retention of all participants over the duration of the study, which we believe was attributable to the interviewer’s skill. Given the growing appreciation of the episodic nature of other illnesses, this methodology may be of interest to others, with the aim to understand the challenges of dealing with fluctuations in health and disability.

This study has a number of limitations. There is some conceptual overlap between the identified phenotypes among participants. For example, many participants experienced stability in some of their health challenges but not others. Given the multiple comorbidities and associated disability, it may not be possible to identify completely discrete phenotypes. While to our knowledge this is the first study to examine episodic disability longitudinally, the 20-month time frame may not have been sufficient for fluctuations in disability to occur in some participants. Nonetheless, this study serves to highlight the complex nature of the disability experienced by PLWH and the challenges associated with managing multiple conditions that may be unpredictable.

This work builds on others by identifying the varied trajectories of episodic disability experienced by PLWH over time. The complexity of the participants’ disability experiences was highlighted through the longitudinal analyses. Although some experienced significant fluctuations and increased disability over time, others were stable or improved during the course of the study. Understanding a person’s episodic trajectory may help to tailor interventions to promote stability, mitigate an upward trajectory of increasing disability, and increase the time between episodes of illness. Further research should focus on understanding the triggers of episodes of illness, and the way in which contextual factors may exacerbate or alleviate disability, and evaluating strategies such as those in self-management interventions to prolong the time between episodes, and mitigate disability.
